# Der Einfluss postmigratorischer Stressoren auf die Prävalenz depressiver Symptome bei Geflüchteten in Deutschland. Analyse anhand der IAB-BAMF-SOEP-Befragung 2016

**DOI:** 10.1007/s00103-020-03238-0

**Published:** 2020-10-25

**Authors:** Niklas Nutsch, Kayvan Bozorgmehr

**Affiliations:** 1 AG2 – Bevölkerungsmedizin und Versorgungsforschung, Fakultät für Gesundheitswissenschaften, Universität Bielefeld, Postfach 10 01 31, 33501 Bielefeld, Deutschland; 2 Abteilung Allgemeinmedizin und Versorgungsforschung, Sektion Health Equity Studies & Migration, Im Neuenheimer Feld 130.3, 69120 Heidelberg, Deutschland

**Keywords:** Asylsuchende, Flüchtlinge, Psychische Gesundheit, Depression, Postmigrationsstressoren, Asylum seekers, Refugees, Mental health, Depression, Postmigration stress

## Abstract

**Einleitung:**

Internationale Studien belegen eine hohe psychische Krankheitslast bei Geflüchteten. Postmigratorische Stressoren im Zufluchtsland können die psychische Gesundheit beeinträchtigen und zu Depressionen führen.

**Ziel:**

Die Studie untersucht, ob postmigratorische Stressoren mit der Prävalenz depressiver Symptome bei erwachsenen Geflüchteten in Deutschland assoziiert sind.

**Methoden:**

Sekundärdatenanalyse basierend auf für Deutschland repräsentativen Querschnittsdaten der IAB-BAMF-SOEP-Befragung von Geflüchteten 2016 (*N* = 4465). Depressivität wurde mit dem Patient Health Questionnaire‑2 (PHQ-2) erfasst. Unadjustierte und adjustierte Odds Ratios (OR) und 95 %-Konfidenzintervalle (KI) wurden anhand binär logistischer Regressionsmodelle berechnet, um Zusammenhänge zwischen Depressivität und soziodemografischen, postmigratorischen und psychosozialen Faktoren zu untersuchen.

**Ergebnisse:**

Depressive Symptome weisen 19,4 % der befragten Geflüchteten auf. Fast alle einbezogenen Postmigrationsstressoren sind nach Adjustierung für soziodemografische und psychosoziale Faktoren statistisch signifikant mit Depressivität assoziiert. Erwerbslosigkeit (aOR = 1,48 [1,04–2,12]), Einsamkeit (aOR = 1,14 [1,10–1,18]) und ein abgelehnter oder noch nicht entschiedener Asylantrag (aOR = 1,34 [1,06–1,70]) erhöhen die Chance für depressive Symptome, während eine stattgefundene Anhörung (aOR = 0,71 [0,56–0,91]) und eine höhere Wohnzufriedenheit (aOR = 0,94 [0,91–0,98]) die Chance für Depressivität verringern.

**Diskussion:**

Postmigrationsstressoren bei Geflüchteten sind mit dem Vorkommen depressiver Symptome assoziiert. Die Berücksichtigung belastender und protektiver Faktoren der Postmigrationsphase in sozialpolitischen Maßnahmen kann die psychische Krankheitslast in Flüchtlingspopulationen reduzieren.

**Zusatzmaterial online:**

Zusätzliche Informationen sind in der Online-Version dieses Artikels (10.1007/s00103-020-03238-0) enthalten.

## Abstract (en)

**Background:**

International studies prove the high burden of mental illnesses among refugees. Postmigration stressors in the country of refuge may affect the mental health and result in depression.

**Objectives:**

The study examines whether postmigration stressors are associated with the prevalence of depressive symptoms among adult refugees in Germany.

**Methods:**

Secondary data analysis based on cross-sectional data of the IAB-BAMF-SOEP Survey of Refugees 2016 (*N* = 4465) that is representative for Germany. Depressive symptoms were measured using the Patient Health Questionnaire‑2 (PHQ-2). Unadjusted and adjusted odds ratios (ORs) and confidence intervals (CIs) were calculated with binary logistic regression models to examine associations between depression and sociodemographic, postmigration, and psychosocial factors.

**Results:**

Depressive symptoms show 19.4% of the surveyed refugees. Almost all selected postmigration stressors are significantly associated with depression after adjustment for sociodemographic and psychosocial factors. Unemployment (aOR = 1.48 [1.04–2.12]), loneliness (aOR = 1.14 [1.10–1.18]), and a rejected or undecided asylum application (aOR = 1.34 [1.06–1.70]) increase the odds of depressive symptoms, whereas asylum interviews (aOR = 0.71 [0.56–0.91]) and higher housing satisfaction (aOR = 0.94 [0.91–0.98]) lower the odds of depression.

**Conclusions:**

Postmigration stress is associated with the occurrence of depressive symptoms. Sociopolitical interventions considering stressful and protective factors of the postmigration phase can reduce the burden of mental disorders in refugee populations.

## Einleitung

Weltweit ist die Anzahl der Menschen, die auf der Flucht vor Verfolgung, Krieg, Gewalt und massiven Menschenrechtsverletzungen sind, im vergangenen Jahrzehnt deutlich angestiegen [1]. Mit mehr als einer Million Asylanträgen hat die Zahl der Asylsuchenden in Deutschland insbesondere in den Jahren 2015 und 2016 stark zugenommen [2, 3]. Jenseits kurzfristiger Schwankungen stellt die Zuwanderung von Flüchtlingen das deutsche Gesundheitssystem vor große strukturelle Herausforderungen [3, 4]. Es besteht eine starke Diskrepanz zwischen dem Bedarf und den tatsächlich existierenden psychotherapeutischen und psychosozialen Versorgungsstrukturen. Das geringe Angebot und der restriktive Zugang zur Gesundheitsversorgung durch das Asylbewerberleistungsgesetz (AsylbLG) verhindern eine frühzeitige Behandlung der Geflüchteten [3–5]. Epidemiologische Untersuchungen belegen eine höhere Prävalenz von psychischen Erkrankungen wie posttraumatische Belastungsstörungen (PTBS), Depressionen oder Angststörungen bei Flüchtlingen im Vergleich zur Bevölkerung der westlichen Industrieländer [6]. Eine umfangreiche Metaanalyse von Steel et al. (2009) zeigt bei Flüchtlingen Prävalenzraten von 30,6 % für die PTBS und 30,8 % für die Depression [7]. In den integrierten Einzelstudien des systematischen Reviews von Bogic et al. (2015) variieren die Prävalenzraten der Depression zwischen 2,3 % und 80 % und die der PTBS zwischen 4,4 % und 86 % [8].

Jedoch können nicht nur existenzbedrohliche Zustände im Herkunftsland und traumatisierende Ereignisse während der Flucht, sondern auch die Lebensbedingungen im Aufnahmeland die psychische Gesundheit von Flüchtlingen beeinträchtigen. Während kriegsbezogene Faktoren starke Prädiktoren für die Entwicklung der PTBS darstellen, sind postmigratorische Faktoren eher mit depressiven Störungen, Angststörungen und Substanzmissbrauch assoziiert [9]. Die Postmigrationsphase ist durch eine Vielzahl an Belastungsfaktoren gekennzeichnet, die einzeln oder kombiniert auftreten können [6, 10]. Eine niederländische Studie von Laban et al. (2005) identifizierte 5 Cluster von Postmigrationsstressoren: familienbezogene Aspekte, Diskriminierung, Asylverfahren, sozioökonomische Lebensbedingungen und sozioreligiöse Aspekte. Diese Cluster stehen in einem signifikanten Zusammenhang mit depressiven Störungen. Sorgen bezüglich des Asylverfahrens, Arbeitslosigkeit und familienbezogene Probleme sind am stärkstem mit der Psychopathologie von Asylsuchenden assoziiert [11].

Bisherige Studien zur psychischen Gesundheit von Geflüchteten basieren häufig auf geringen, nicht repräsentativen Fallzahlen und sind regional begrenzt. Trotz der hohen Anzahl an Studien verhindert die starke Heterogenität in Bezug auf Studienpopulationen, Erhebungsinstrumente, Settings, Sampling-Strategien und Klassifikationen die Vergleichbarkeit der Ergebnisse. Dadurch bleibt der Erkenntnisgewinn moderat [3, 12]. Es besteht ein zentraler Bedarf an repräsentativen Untersuchungen zum psychischen Gesundheitszustand von Flüchtlingen in Deutschland [3, 9, 13]. Demzufolge untersucht die vorliegende Studie anhand einer repräsentativen bundesweiten Stichprobe, ob postmigratorische Stressoren bei erwachsenen Geflüchteten nach der Kontrolle von Unterschieden in soziodemografischen und psychosozialen Merkmalen mit dem Vorkommen depressiver Symptome assoziiert sind.

## Methoden

### Datensatz

Die Sekundärdatenanalyse basiert auf den Daten einer Befragung von Geflüchteten aus dem Jahr 2016, die von der Forschungskooperation zwischen dem Institut für Arbeitsmarkt- und Berufsforschung (IAB), dem Forschungszentrum Migration, Integration und Asyl des Bundesamtes für Migration und Flüchtlinge (BAMF-FZ) und dem Sozio-oekonomischen Panel (SOEP) am Deutschen Institut für Wirtschaftsforschung (DIW Berlin) durchgeführt wurde (IAB-BAMF-SOEP-Befragung). Geflüchtete, die zwischen dem 01.01.2013 und dem 31.01.2016 nach Deutschland eingereist sind und einen Asylantrag beim Bundesamt für Migration und Flüchtlinge (BAMF) gestellt haben oder im Rahmen spezieller Programme auf Bundes- oder Landesebene aufgenommen wurden, bilden die Grundgesamtheit der Stichprobe.

Die Stichprobe besteht aus 2 Unterstichproben und basiert auf einem stratifizierten mehrstufig geclusterten Sampling-Design. Die Datenbasis für die Ziehung der Zufallsstichprobe bildet das Ausländerzentralregister (AZR). Zur Berücksichtigung der verzögerten Erfassung der eingereisten Schutzsuchenden, die im Jahr 2015 nicht direkt im Anschluss an die Einreise einen Asylantrag stellen konnten, wurde die Stichprobenziehung in 4 zeitlich versetzte Tranchen aufgeteilt. Dabei wurden überproportional viele Frauen und Personen im Alter von über 30 Jahren berücksichtigt, um ausreichend Fallzahlen für diese relativ kleinen Gruppen der Grundgesamtheit zu erhalten. Durch geeignete Gewichtungsverfahren sind die Unterstichproben und alle Tranchen zusammen analysierbar.

Befragt wurden alle näheren erwachsenen Familienmitglieder desselben Haushaltes einer aus dem AZR gezogenen Befragungsperson (Ankerperson). Unbegleitete Kinder und Jugendliche wurden aus forschungsethischen und datenschutzrechtlichen Gründen von der Stichprobenziehung ausgeschlossen. Bei 9902 kontaktierten Ankerpersonen sind 3054 qualitätsneutrale Drop-outs (z. B. ungültige Adressen) gegeben. Von den übrigen 6848 Ankerpersonen haben 3336 an der Befragung teilgenommen. Somit beträgt die Rücklaufquote 48,6 %. Insgesamt wurden 4527 erwachsene Geflüchtete interviewt.

Für die Datenerhebung haben 290 geschulte Personen des Instituts KANTAR Public von Ende Juni bis Ende Dezember 2016 computerunterstützte persönliche Face-to-Face-Interviews in Erstaufnahmeeinrichtungen, Sammelunterkünften und Privathaushalten in 169 Befragungsregionen durchgeführt, die repräsentativ über das Bundesgebiet verteilt sind. Die statistischen Analysen basieren auf dem integrierten Personen- und Biografiefragebogen, der etwa 450 Fragen enthält. Die Erhebungsinstrumente standen den Befragten schriftlich und mündlich in 7 Sprachen (Arabisch, Kurmandschi, Farsi/Dari, Urdu, Paschtu, Deutsch, Englisch) zur Verfügung und wurden für Teilnehmende mit geringer Lesekompetenz durch eingesprochene Audiodateien unterstützt. In den Interviews wurden professionelle (1,7 %) und informelle (34,1 %) Dolmetscher eingesetzt. Die Befragten wurden über die Befragung aufgeklärt und informiert, dass die Befragung freiwillig und unabhängig von rechtlichen Verfahren ist. Die Befragung wurde von dem Survey Committee des SOEP genehmigt und durchlief eine juristische Prüfung durch das BAMF [14–17].

### Messung der Depressivität (abhängige Variable)

Die Depressivität wurde mit dem Patient Health Questionnaire‑2 (PHQ-2) gemessen. Dieses Selbstbeurteilungsverfahren zum Screening der Depressivität erfasst mit 2 Items die Kernsymptome der Majordepression nach dem Diagnostic and Statistical Manual of Mental Disorders (DSM-IV). Die beiden Items nehmen addiert einen Wert von 0 bis 6 an. Ab einem Punktwert von 3 oder höher gilt das Testergebnis als auffällig und das Vorliegen einer depressiven Symptomatik sollte durch eine anschließende Diagnostik abgeklärt werden. In Orientierung an diesem Testergebnis wurde eine binär codierte Outcomevariable erstellt (keine Depressivität; Depressivität) [18, 19].

### Messung der soziodemografischen Faktoren (unabhängige Variablen)

Das Alter der Befragten wurde anhand einer metrischen Variablen, das Geschlecht anhand einer dichotomen Variablen (männlich; weiblich) in die Analysen einbezogen. Zusätzlich wurde eine dichotome Variable zur Partnerschaft mit den Kategorien „Ja“ (verheiratet) und „Nein“ (ledig; geschieden; verwitwet) erstellt. Für die Beurteilung des Bildungsstands wurden 2 Variablen zum letzten Schulbesuch im Ausland umcodiert und zu einer ordinalen Variablen mit 4 Kategorien zusammengefasst (kein Schulbesuch; Grundschule; Mittelschule; weiterführende/andere Schule). Außerdem wurden die Daten der IAB-BAMF-SOEP-Befragung von Geflüchteten durch das Heranziehen externer Daten zur Political Terror Scale (PTS) ergänzt [20]. Diesbezüglich konnten die Staatsangehörigkeiten der Geflüchteten den 5 Werten der PTS zugeordnet werden. Die PTS zielt auf die Messung des politischen Terrors ab und umfasst etwa 200 Staaten und Gebiete von 1976 bis 2018. Die Zuordnung basiert auf Informationen der Organisation Amnesty International. Für die Codierung wurden Angaben aus dem Jahr 2016 genutzt. Höhere Werte der PTS beschreiben ein größeres Ausmaß an politischem Terror [20].

### Messung der postmigratorischen Stressoren (unabhängige Variablen)

Die Variable zum Aufenthaltsstatus umfasst die 3 Kategorien „Anerkennung“ (Flüchtlingsstatus; Asylberechtigte; anderer Schutzstatus), „Ablehnung“ (Duldung; Ausreiseaufforderung) und „Asylsuchende“. Die dichotomisierte Variable zur Anhörung (Ja; Nein) enthält Informationen darüber, ob eine offizielle Anhörung des Asylantrags beim BAMF stattgefunden hat oder nicht. Zudem wurde eine Variable zur aktuellen Erwerbstätigkeit dichotomisiert (erwerbstätig; nicht erwerbstätig). Die Variablen zur gegenwärtigen Wohnzufriedenheit basieren auf einer 11-stufigen bipolaren Ratingskala von 0 (ganz und gar unzufrieden) bis 10 (ganz und gar zufrieden). Um die deutschen Sprachkenntnisse zu ermitteln, sollten die Geflüchteten auf einer verbalen Schätzskala beurteilen, wie gut sie die deutsche Sprache sprechen, schreiben und lesen können. Neben einer metrischen Variablen wurde ein Index mit den Kategorien „gut“, „mittelmäßig“ und „schlecht“ erstellt, der die Antwortmöglichkeiten der 3 Items sinnvoll kombiniert. Die Erfassung von Einsamkeitsgefühlen erfolgte mit den 3 Items der Loneliness Scale-SOEP (LS‑S). Es wurde eine metrische Variable berechnet, die einen Skalengesamtwert von 0 bis 12 wiedergibt. Je höher der Gesamtscore, desto höher auch die Einsamkeit. Ergänzend wurde ein Index mit den Kategorien „niedrig“, „mittelmäßig“ und „hoch“ erstellt [19].

### Messung der psychosozialen Faktoren (unabhängige Variablen)

Die allgemeine Lebenszufriedenheit wurde mit der Single-Item-Skala Lebenszufriedenheit‑1 (L-1) auf einer Ratingskala von 0 (ganz und gar unzufrieden) bis 10 (ganz und gar zufrieden) beurteilt. Die kategorisierte Variable enthält die Kategorien „hoch“ (Skalenwert 7–10), „mittelmäßig“ (Skalenwert 4–6) und „gering“ (Skalenwert 0–3; [21]). Auch das Selbstwertgefühl konnte mit nur einem Item auf einer bipolaren Ratingskala eingeschätzt werden. Dazu wurde eine Variable mit den 3 Kategorien „hoch“ (Skalenwert 6–7), „mittelmäßig“ (Skalenwert 3–5) und „gering“ (Skalenwert 1–2) erstellt. Für die metrische Variable wurde die 7‑stufige Skala in Orientierung an die Originalskala zu einer 5‑stufigen Skala umcodiert [22, 23]. Die Messung des resilienten Copingverhaltens bzw. des resilienten Copingstils erfolgte mit der Brief Resilient Coping Scale (BRCS). Vier Items wurden auf 7‑stufigen bipolaren Ratingskalen beurteilt, die in Orientierung an der Originalskala zu 5‑stufigen Skalen umcodiert wurden. Die berechnete metrische Variable kann einen Gesamtscore von 4 bis 20 annehmen. Cut-off-Werte wurden genutzt, um eine Variable mit den Kategorien „gering“ (Skalengesamtwert 4–13), „mittelmäßig“ (Skalengesamtwert 14–16) und „hoch“ (Skalengesamtwert 17–20) zu erstellen [19, 24]. Ängstlichkeit wurde mit dem Generalized Anxiety Disorder‑2 (GAD-2) gemessen. Das Testverfahren besteht aus 2 Items, die sich von den Kernsymptomen der generalisierten Angststörung ableiten und addiert einen Wert von 0 bis 6 annehmen. Ab einem Punktwert von 3 oder höher gilt das Testergebnis als auffällig und ist ein ernstzunehmender Hinweis auf das Vorliegen einer Angststörung [19].

### Statistische Analysen

Die statistischen Analysen wurden mit IBM SPSS Statistics 25 durchgeführt. Alle statistischen Tests basieren auf einem Signifikanzniveau α von 0,05. In den deskriptiven Analysen wurden für die nominal- und ordinalskalierten Variablen absolute und prozentuale Häufigkeiten untersucht, während für die metrischen Variablen Werte der zentralen Tendenz (Mittelwerte) und Streuungsmaße (Standardabweichungen) berechnet wurden. Diese deskriptiven Analysen erfolgten ohne Gewichtung. Für die anschließenden inferenzstatistischen Verfahren wurde ein Hochrechnungsfaktor für die Grundgesamtheit der Geflüchteten in Deutschland aktiviert. Nach der Prüfung von mehreren Voraussetzungen wurden binär logistische Regressionsanalysen durchgeführt. Mit dem Box-Tidwell-Test erfolgte für alle metrischen unabhängigen Variablen ein Test auf Linearität im Logit. Zusätzlich zeigt eine Multikollinearitätsdiagnose, dass die Toleranzwerte den Grenzwert von 0,25 nicht unterschreiten und die Varianz-Inflations-Faktoren (VIF) den Grenzwert von 4,00 nicht überschreiten. Es wurden unadjustierte Regressionsmodelle berechnet, um bivariate Zusammenhänge zwischen der abhängigen und den einzelnen unabhängigen Variablen zu ermitteln. Für die 3 Variablengruppen zu den soziodemografischen, postmigratorischen und psychosozialen Faktoren wurden adjustierte Regressionsmodelle berechnet (Modell I, II und III). Zusätzlich wurde ein adjustiertes Regressionsmodell mit den ersten beiden Variablengruppen (Modell IV) und ein adjustiertes Gesamtmodell mit allen Variablengruppen (Modell V) erstellt. Die Entwicklung der adjustierten Regressionsmodelle erfolgte mit einem schrittweisen Einschluss der einzelnen unabhängigen Variablen (siehe Online-Zusatzmaterial zu diesem Beitrag). Für die Prädiktorvariablen wurden Odds Ratios (OR) mit 95 %-Konfidenzintervallen (KI) berechnet. Die Modellgüte wurde in Orientierung an Nagelkerkes R^2^ evaluiert [25].

## Ergebnisse

### Ergebnisse der deskriptiven Analysen

Von 4465 Geflüchteten der IAB-BAMF-SOEP-Befragung lagen für 4136 (92,6 %) Personen gültige Werte des PHQ‑2 bzw. zur Depressivität vor. Davon weisen 19,4 % ein auffälliges Testergebnis auf.

Mehr als die Hälfte der Befragten sind 18 bis 35 Jahre alt (61,0 %)(Tab. 1). Das durchschnittliche Alter beträgt 33,6 Jahre. Die untersuchte Stichprobe ist überwiegend männlich (62,1 %) und mehr als die Hälfte (65,9 %) berichten, dass sie verheiratet sind. 62,4 % der Geflüchteten haben außerhalb von Deutschland die Mittelschule oder eine weiterführende Schule besucht, während 10,5 % keine Schule besucht haben. Die untersuchte Stichprobe besteht zu 90 % aus Personen, deren Staatsangehörigkeiten den beiden höchsten Werten 4 und 5 der Political Terror Scale (PTS) zugeordnet sind. Am häufigsten geben die Befragten die syrische (49,0 %), irakische (12,9 %) und afghanische (12,8 %) Staatsangehörigkeit an (nicht dargestellt).

**Table 1:** 

		Gesamt *N* (%)	Depressivität nach PHQ‑2			
			Keine Depressivität *N* (%)	Depressivität *N* (%)	Gesamt *N* (%)	Fehlend *N* (%)
Soziodemografische Faktoren						
Alter	18 bis 25 Jahre	1097 (24,6)	807 (24,7)	210 (24,3)	1017 (24,6)	–
	26 bis 35 Jahre	1627 (36,4)	1219 (37,3)	274 (31,7)	1493 (36,1)	–
	36 bis 45 Jahre	1130 (25,3)	819 (25,0)	239 (27,7)	1058 (25,6)	–
	46 bis 55 Jahre	468 (10,5)	332 (10,1)	109 (12,6)	441 (10,7)	–
	>55 Jahre	143 (3,2)	95 (2,9)	32 (3,7)	127 (3,1)	–
	Mittelwert (SD)	33,6 (10,4)	33,4 (10,2)	34,3 (10,8)	–	–
	*N* (%)	4465 (100)	3272 (100)	864 (100)	4136 (100)	329 (7,4)
Geschlecht	Männlich	2773 (62,1)	2058 (62,9)	526 (60,9)	2584 (62,5)	–
	Weiblich	1692 (37,9)	1214 (37,1)	338 (39,1)	1552 (37,5)	–
	*N* (%)	4465 (100)	3272 (100)	864 (100)	1552 (100)	329 (7,4)
Familienstand	Ledig	1298 (29,1)	956 (29,4)	259 (30,2)	1215 (29,5)	–
	Verheiratet	2942 (65,9)	2175 (66,8)	554 (64,6)	2729 (66,4)	–
	Geschieden	101 (2,3)	75 (2,3)	17 (2,0)	92 (2,2)	–
	Verwitwet	89 (2,0)	49 (1,5)	28 (3,3)	77 (1,9)	–
	*N* (%)	4430 (99,2)	3255 (100)	858 (100)	4113 (100)	352 (7,9)
Bildung (Schulbesuch im Ausland)	Kein Schulbesuch	469 (10,5)	309 (10,3)	117 (14,7)	426 (11,2)	–
	Grundschule	655 (14,7)	470 (15,7)	130 (16,4)	600 (15,8)	–
	Mittelschule	1287 (28,8)	975 (32,5)	222 (27,9)	1197 (31,6)	–
	Weiterführende Schule	1502 (33,6)	1154 (38,5)	295 (37,1)	1449 (38,2)	–
	Andere Schule	127 (2,8)	89 (3,0)	31 (3,9)	120 (3,2)	–
	*N* (%)	4040 (90,5)	2997 (100)	795 (100)	3792 (100)	673 (15,1)
PTS-Wert der Staatsangehörigkeit	PTS-Wert 1	123 (2,8)	86 (2,7)	27 (3,2)	113 (2,8)	–
	PTS-Wert 2	135 (3,0)	92 (2,9)	38 (4,5)	130 (3,2)	–
	PTS-Wert 3	116 (2,6)	79 (2,5)	24 (2,8)	103 (2,5)	–
	PTS-Wert 4	570 (12,8)	413 (12,8)	95 (11,2)	508 (12,5)	–
	PTS-Wert 5	3447 (77,2)	2551 (79,2)	662 (78,3)	3213 (79,0)	–
	*N* (%)	4391 (98,3)	3221 (100)	846 (100)	4067 (100)	398 (8,9)
Postmigratorische Stressoren						
Aufenthaltsstatus	Asylsuchende	1505 (33,7)	1025 (33,2)	347 (42,5)	1372 (35,1)	–
	Asylberechtigte/Flüchtlingsstatus	2256 (50,5)	1752 (56,7)	369 (45,2)	2121 (54,3)	–
	Anderer Schutzstatus	200 (4,5)	160 (5,2)	34 (4,2)	194 (5,0)	–
	Duldung	163 (3,7)	102 (3,3)	47 (5,8)	149 (3,8)	–
	Ausreiseaufforderung	76 (1,7)	50 (1,5)	20 (2,4)	70 (1,8)	–
	*N* (%)	4200 (94,1)	3089 (100)	817 (100)	3906 (100)	559 (12,5)
Anhörung	Nein	894 (20,0)	639 (20,7)	197 (24,1)	836 (21,4)	–
	Ja	3289 (73,7)	2446 (79,3)	620 (75,9)	3066 (78,6)	–
	*N* (%)	4183 (93,7)	3085 (100)	817 (100)	3902 (100)	563 (12,6)
Erwerbstätigkeit	Vollzeit	138 (3,1)	112 (3,4)	18 (2,1)	130 (3,1)	–
	Teilzeit/Geringfügig	214 (4,8)	163 (5,0)	38 (4,4)	201 (4,9)	–
	Ausbildung/Praktikum	131 (2,9)	105 (3,2)	15 (1,7)	120 (2,9)	–
	Keine	3982 (89,2)	2892 (88,4)	793 (91,8)	3685 (89,1)	–
	*N* (%)	4465 (100)	3272 (100)	864 (100)	4136 (100)	329 (7,4)
Wohnzufriedenheit	Hoch	2586 (57,9)	2014 (61,8)	379 (44,2)	2393 (58,1)	–
	Mittelmäßig	1189 (26,6)	840 (25,8)	275 (32,1)	1115 (27,1)	–
	Niedrig	663 (14,8)	406 (12,5)	203 (23,7)	609 (14,8)	–
	Mittelwert (SD)	6,7 (3,0)	6,9 (2,8)	5,7 (3,2)	–	–
	*N* (%)	4438 (99,4)	3260 (100)	857 (100)	4117 (100)	348 (7,8)
Sprachkenntnisse (Deutsch)	Gut	899 (20,1)	724 (22,2)	131 (15,2)	855 (20,7)	–
	Mittelmäßig	1411 (31,6)	1063 (32,5)	259 (30,0)	1332 (32,0)	–
	Schlecht	2151 (48,2)	1481 (45,3)	474 (54,9)	1955 (47,3)	–
	Mittelwert (SD)	10,2 (2,9)	10,0 (2,9)	10,7 (2,9)	–	–
	*N* (%)	4461 (99,9)	3268 (100)	864 (100)	4132 (100)	333 (7,5)
Einsamkeit (LS-S)	Niedrig	2022 (45,3)	1699 (54,6)	236 (29,1)	1935 (49,3)	–
	Mittelmäßig	1191 (26,7)	886 (28,5)	243 (30,0)	1129 (28,8)	–
	Hoch	898 (20,1)	529 (17,0)	331 (40,9)	860 (21,9)	–
	Mittelwert (SD)	4,7 (3,3)	4,3 (3,1)	6,3 (3,3)	–	–
	*N* (%)	4111 (92,1)	3114 (100)	810 (100)	3924 (100)	541 (12,1)
Psychosoziale Faktoren						
Selbstwertgefühl	Hoch	3269 (73,2)	2535 (82,4)	586 (72,9)	3121 (80,4)	–
	Mittelmäßig	737 (16,5)	508 (16,5)	187 (23,3)	695 (17,9)	–
	Gering	70 (1,6)	34 (1,1)	31 (3,9)	65 (1,7)	–
	Mittelwert (SD)	4,5 (0,8)	4,6 (0,7)	4,4 (1,0)	–	–
	*N* (%)	4076 (91,3)	3077 (100)	804 (100)	3881 (100)	584 (13,1)
Allgemeine Lebenszufriedenheit (L-1)	Hoch	2991 (67,0)	2371 (72,7)	421 (49,0)	2792 (67,8)	–
	Mittelmäßig	1143 (25,6)	767 (23,5)	297 (34,5)	1064 (25,8)	–
	Gering	288 (6,5)	123 (3,8)	142 (16,6)	265 (6,4)	–
	Mittelwert (SD)	7,2 (2,3)	7,6 (2,1)	6,2 (2,8)	–	–
	*N* (%)	4422 (99,0)	3261 (100)	860 (100)	4121 (100)	344 (7,7)
Resilientes Copingverhalten (BRCS)	Hoch	2651 (59,4)	2036 (70,7)	508 (66,8)	2544 (69,9)	–
	Mittelmäßig	902 (20,2)	707 (24,6)	173 (22,8)	880 (24,2)	–
	Gering	223 (5,0)	136 (4,7)	79 (10,4)	215 (5,9)	–
	Mittelwert (SD)	17,7 (2,4)	17,8 (2,3)	17,3 (2,7)	–	–
	*N* (%)	3776 (84,6)	2879 (100)	760 (100)	3639 (100)	826 (18,5)
Ängstlichkeit (GAD-2)	Ängstlichkeit	962 (21,5)	406 (12,6)	510 (61,2)	916 (22,6)	–
	Keine Ängstlichkeit	3237 (72,5)	2805 (87,4)	324 (38,8)	3129 (77,4)	–
	Mittelwert (SD)	1,6 (1,7)	1,2 (1,3)	3,2 (1,9)	–	–
	*N* (%)	4199 (94,0)	3211 (100)	834 (100)	4045 (100)	420 (9,4)

*N* absolute Häufigkeiten der untersuchten Personen, Variationen in *N* resultieren aus fehlenden Werten, *SD* Standardabweichung des Mittelwertes, *PHQ‑2* Patient Health Questionnaire‑2, *PTS* Political Terror Scale, *LS‑S* Loneliness Scale-SOEP, *L‑1* Lebenszufriedenheit‑1, *BRCS* Brief Resilient Coping Scale, *GAD‑2* Generalized Anxiety Disorder‑2

Die Personengruppe mit Depressivität weist weniger häufig einen anerkannten Aufnahmestatus (asylberechtigt, Flüchtlingsstatus, anderer Schutzstatus) auf (49,4 %) als die Personengruppe ohne Depressivität (61,9 %)(Tab. 1). Bei 42,5 % der Depressiven und bei 33,2 % der Nichtdepressiven wurde noch nicht über den Asylantrag entschieden. Bei 73,7 % aller Befragten hat eine offizielle Anhörung des Asylantrags stattgefunden. Ein Großteil der Befragten gibt an, nicht erwerbstätig zu sein (89,2 %). In Bezug auf die Wohnsituation berichten 44,2 % der Personen mit Depressivität und 61,8 % der Personen ohne Depressivität eine hohe Zufriedenheit. Fast die Hälfte aller Befragten schätzen ihre deutschen Sprachkenntnisse als schlecht ein (48,2 %). Die Gruppe der Depressiven weist mit einem höheren Mittelwert auf der LS‑S mehr Einsamkeitsgefühle auf. 40,9 % der Geflüchteten mit Depressivität und 17,0 % der Geflüchteten ohne Depressivität berichten eine hohe Einsamkeit.

73,2 % aller Befragten geben ein hohes Selbstwertgefühl an (Tab. 1). Mit einem Mittelwert von 6,2 auf der Kurzskala L‑1 ist die allgemeine Lebenszufriedenheit bei Geflüchteten mit Depressivität geringer als bei Geflüchteten ohne Depressivität, die einen Mittelwert von 7,6 ausweisen. Für fast 60 % aller Befragten konnte ein hohes resilientes Copingverhalten ermittelt werden. In der Gruppe der Depressiven zeigen 10,4 % einen geringen resilienten Copingstil, während es in der Gruppe der Nichtdepressiven 4,7 % sind. Bei 21,5 % der Befragten besteht ein ernst zu nehmender Hinweis auf eine Angststörung. 61,2 % der Depressiven und 12,6 % der Nichtdepressiven weisen pathologische Ängstlichkeit auf.

### Ergebnisse der bivariaten und multivariaten Analysen

#### Zusammenhang zwischen soziodemografischen Faktoren und Depressivität

Die bivariaten Modelle zu den soziodemografischen Faktoren zeigen ausschließlich für die Bildung statistisch signifikante Zusammenhänge (Tab. 2). Geflüchtete, die außerhalb von Deutschland eine Grundschule (OR = 0,68 [0,51–0,92]), eine Mittelschule (OR = 0,68 [0,53–0,88]) oder eine weiterführende bzw. eine andere Schule (OR = 0,75 [0,59–0,96]) besucht haben, weisen eine geringere Chance für Depressivität auf als Geflüchtete ohne Schulbesuch. Nach Adjustierung der soziodemografischen Faktoren in Modell I weichen die Effektkoeffizienten nur geringfügig von denen der bivariaten Modelle ab.

**Table 2:** 

Prädiktorvariablen OR [95%-KI]		Bivariate Modelle	*p*-Wert	Multivariates Modell
				Modell I
*Alter*	–	1,005 [0,998–1,012]	0,146	**1,010 [1,001–1,018]**
*Geschlecht*	Männlich	1 *(Ref.)*	–	1 *(Ref.)*
	Weiblich	1,003 [0,852–1,179]	0,974	1,053 [0,876–1,267]
*Partnerschaft*	Ja	1 *(Ref.)*	–	1 *(Ref.)*
	Nein	1,132 [0,979–1,308]	0,094	1,188 [0,989–1,426]
*Bildung*	Kein Schulbesuch	1 *(Ref.)*	–	1 *(Ref.)*
	Grundschule	**0,684 [0,508–0,921]**	0,012	**0,661 [0,488–0,894]**
	Mittelschule	**0,678 [0,525–0,877]**	0,003	**0,680 [0,523–0,886]**
	Weiterführende/ andere Schule	**0,751 [0,589–0,957]**	0,021	**0,713 [0,555–0,916]**
*PTS-Wert der Staatsangehörigkeit*	PTS-Wert 1–3	1 *(Ref.)*	–	1 *(Ref.)*
	PTS-Wert 4–5	0,978 [0,793–1,206]	0,833	1,081 [0,857–1,363]
*Nagelkerkes R*^2^	–	–	–	0,007
*N*	–	–	–	3711

*OR* Odds Ratios verglichen mit der Referenzgruppe, *KI* 95 %-Konfidenzintervalle, *N* absolute Häufigkeiten der untersuchten Personen, Variationen in *N* resultieren aus fehlenden Werten

*Depressivität*: Patient Health Questionnaire‑2 (PHQ-2), keine Depressivität = Score 0–2, Depressivität = Score 3–6; *Partnerschaft*: Ja = verheiratet, Nein = ledig, geschieden, verwitwet; *Staatsangehörigkeit*: PTS-Wert 1–3 = Wert 1 bis 3 auf der Political Terror Scale, PTS-Wert 4–5: Wert 4 oder 5 auf der Political Terror Scale; *fett gedruckte Zahlen*: Odds Ratios größer oder kleiner 1 mit einem Konfidenzniveau von 95 %; gewichtete Ergebnisse

#### Zusammenhang zwischen postmigratorischen Stressoren und Depressivität

In den bivariaten Analysen konnten für alle ausgewählten postmigratorischen Stressoren statistisch signifikante Zusammenhänge mit Depressivität ermittelt werden (Tab. 3). Geflüchtete mit einem abgelehnten oder noch nicht entschiedenen Asylantrag haben eine 1,76-fach [1,52–2,05] höhere Chance, depressive Symptome aufzuweisen, als Geflüchtete mit einem anerkannten Asylantrag. Im Vergleich zur Referenzgruppe weisen Personen, bei denen eine offizielle Anhörung stattgefunden hat, eine geringere Chance für depressive Symptome auf (OR = 0,76 [0,65–0,89]). Die bivariaten Analysen zeigen, dass die Chance für Depressivität bei Geflüchteten ohne Erwerbstätigkeit fast doppelt so hoch ist wie bei Geflüchteten mit Erwerbstätigkeit (OR = 1,80 [1,40–2,32]). Personen, die ihre deutschen Sprachkenntnisse schlecht einschätzen, haben im Vergleich zu Personen, die ihre Deutschkenntnisse gut einschätzen, ebenfalls eine fast doppelt so hohe Chance für depressive Symptome (OR = 1,84 [1,50–2,26]). Die Chance für Depressivität reduziert sich mit steigender Wohnzufriedenheit, und zwar pro Anstieg um eine Einheit auf der 11-stufigen Skala zur Wohnzufriedenheit um den Multiplikationsfaktor 0,85 [0,83–0,88]. Das unadjustierte Regressionsmodell zur Einsamkeit zeigt, dass die Chance für depressive Symptome mit jeder Erhöhung um eine Einheit auf der LS‑S um das 1,24-Fache [1,21‑1,27] ansteigt. Nach Adjustierung der postmigratorischen Stressoren in Modell II reduziert sich die Stärke des Zusammenhangs zwischen Depressivität und dem Aufenthaltsstatus, der Anhörung sowie den Sprachkenntnissen und liegt bei den letzteren beiden unterhalb des Signifikanzniveaus.

**Table 3:** 

Prädiktorvariablen OR [95%-KI]		Bivariate Modelle	*p*-Wert	Multivariates Modell
				Modell II
Aufenthaltsstatus	Anerkennung	1 *(Ref.)*	–	1 *(Ref.)*
	Asylsuchend oder Ablehnung	**1,763 [1,516–2,050]**	<0,001	**1,300 [1,087–1,556]**
Anhörung	Nein	1 *(Ref.)*	–	1 *(Ref.)*
	Ja	**0,761 [0,649–0,891]**	0,001	0,933 [0,773–1,127]
Erwerbstätigkeit	Erwerbstätig	1 *(Ref.)*	–	1 *(Ref.)*
	Nicht erwerbstätig	**1,802 [1,402–2,317]**	<0,001	**1,848 [1,365–2,501]**
Wohnzufriedenheit	–	**0,854 [0,834–0,875]**	<0,001	**0,905 [0,881–0,930]**
Sprachkenntnisse (Deutsch)	Gut	1 *(Ref.)*	–	1 *(Ref.)*
	Mittelmäßig	**1,427 [1,147–1,775]**	0,001	1,161 [0,908–1,486]
	Schlecht	**1,838 [1,498–2,255]**	<0,001	1,231 [0,971–1,559]
Einsamkeit	–	**1,238 [1,208–1,269]**	<0,001	**1,207 [1,175–1,240]**
Nagelkerkes R^2^	–	–	–	0,161
*N*	–	–	–	3628

*OR* Odds Ratios verglichen mit der Referenzgruppe, *KI* 95 %-Konfidenzintervalle, *N* absolute Häufigkeiten der untersuchten Personen, Variationen in *N* resultieren aus fehlenden Werten

*Depressivität*: Patient Health Questionnaire‑2 (PHQ-2), keine Depressivität = Score 0–2, Depressivität = Score 3–6; *Aufenthaltsstatus*: Anerkennung = Flüchtlingsstatus, Asylberechtigte, anderer Schutzstatus, asylsuchend = Asylantrag wurde noch nicht entschieden, Ablehnung = Duldung, Ausreiseaufforderung; *Anhörung*: Ja = Anhörung des Asylantrags hat stattgefunden, Nein = Anhörung des Asylantrags hat noch nicht stattgefunden; *Erwerbstätigkeit*: erwerbstätig = Vollzeitbeschäftigung, Teilzeitbeschäftigung, Ausbildung oder Praktikum, nicht erwerbstätig = keine Erwerbstätigkeit; *Wohnzufriedenheit*: Skala von 0 (ganz und gar unzufrieden) bis 10 (ganz und gar zufrieden); *Einsamkeit*: Loneliness Scale-SOEP (LS-S); *fett gedruckte Zahlen*: Odds Ratios größer oder kleiner 1 mit einem Konfidenzniveau von 95 %; gewichtete Ergebnisse

#### Zusammenhang zwischen psychosozialen Faktoren und Depressivität

In den bivariaten Analysen konnten für alle ausgewählten psychosozialen Faktoren statistisch signifikante Zusammenhänge mit Depressivität ermittelt werden (Tab. 4). Die Ergebnisse zeigen, dass Geflüchtete mit einem mittelmäßigen bis geringem Selbstwertgefühl eine 1,64-mal [1,37–1,96] so hohe Chance für Depressivität aufweisen wie Geflüchtete mit einem hohen Selbstwertgefühl. Im Vergleich zu Personen mit einem hohen resilienten Copingstil haben Personen, bei denen ein mittelmäßiges bis geringes resilientes Copingverhalten ermittelt wurde, eine höhere Chance für depressive Symptome (OR = 1,32 [1,12–1,54]). Zusätzlich haben Geflüchtete mit einer geringen allgemeinen Lebenszufriedenheit eine siebenfach höhere Chance, depressiv zu sein, als Geflüchtete mit einer hohen allgemeinen Lebenszufriedenheit (OR = 7,38 [5,88–9,26]). Die Prädiktorvariable zur Ängstlichkeit zeigt die höchsten Effektkoeffizienten. Im unadjustierten Modell haben Personen mit pathologischer Ängstlichkeit eine 12,31-mal [10,38–14,60] so hohe Chance für Depressivität wie Personen ohne pathologische Ängstlichkeit. Nach Adjustierung der psychosozialen Faktoren in Modell III verringern sich alle Effektkoeffizienten und der Zusammenhang zwischen Depressivität und dem Selbstwertgefühl ist nicht mehr statistisch signifikant.

**Table 4:** 

Prädiktorvariablen OR [95%-KI]		Bivariate Modelle	*p*-Wert	Multivariates Modell
				Modell III
*Selbstwertgefühl*	Hoch	1 *(Ref.)*	–	1 *(Ref.)*
–	Mittelmäßig bis gering	**1,639 [1,373–1,956]**	<0,001	1,225 [0,971–1,545]
*Resilientes Copingverhalten*	Hoch	1 *(Ref.)*	–	1 *(Ref.)*
	Mittelmäßig bis gering	**1,318 [1,124–1,544]**	0,001	**1,226 [1,005–1,495]**
*Lebenszufriedenheit*	Hoch	1 *(Ref.)*	–	1 *(Ref.)*
	Mittelmäßig	**1,740 [1,476–2,051]**	<0,001	**1,418 [1,157–1,737]**
	Gering	**7,376 [5,875–9,261]**	<0,001	**3,322 [2,498–4,417]**
*Ängstlichkeit*	Keine Ängstlichkeit	1 *(Ref.)*	–	1 *(Ref.)*
	Ängstlichkeit	**12,310 [10,381–14,599]**	<0,001	**9,799 [8,123–11,821]**
*Nagelkerkes R*^2^	–	–	–	0,330
*N*	–	–	–	3493

*OR* Odds Ratios verglichen mit der Referenzgruppe, *KI* 95 %-Konfidenzintervalle, *N* absolute Häufigkeiten der untersuchten Personen, Variationen in *N* resultieren aus fehlenden Werten

*Depressivität*: Patient Health Questionnaire‑2 (PHQ-2), keine Depressivität = Score 0–2, Depressivität = Score 3–6; *Selbstwertgefühl*: „Ich habe eine positive Einstellung zu mir selbst“, hoch = Skalenwert 6–7, mittelmäßig = Skalenwert 3–5, gering = Skalenwert 1–2; *resilientes Copingverhalten*: Brief Resilient Coping Scale (BRCS), hoch = Score 17–20, mittelmäßig = Score 14–16, gering = Score 4–13; *Lebenszufriedenheit*: Lebenszufriedenheit‑1 (L-1), hoch = Skalenwert 7–10, mittelmäßig = Skalenwert 4–6, gering = Skalenwert 0–3; *Ängstlichkeit*: Generalized Anxiety Disorder‑2 (GAD-2), keine Ängstlichkeit = Score 0–2, Ängstlichkeit = Score 3–6; *fett gedruckte Zahlen*: Odds Ratios größer oder kleiner 1 mit einem Konfidenzniveau von 95 %; gewichtete Ergebnisse

#### Gesamtmodell

Die Ergebnisse der adjustierten Regressionsmodelle zur Untersuchung der multivariaten Zusammenhänge zwischen Depressivität und den soziodemografischen, postmigratorischen und psychosozialen Faktoren sind in Tab. 5 beschrieben.

**Table 5:** 

Prädiktorvariablen OR [95%-KI]		Adjustierte Regressionsmodelle	
		Modell IV	Modell V (Gesamtmodell)
*Soziodemografische Faktoren*			
*Alter*	–	1,006 [0,997–1,016]	1,004 [0,992–1,015]
*Geschlecht*	Männlich	1 *(Ref.)*	1 *(Ref.)*
	Weiblich	1,088 [0,877–1,350]	0,853 [0,653–1,115]
*Partnerschaft*	Ja	1 *(Ref.)*	1 *(Ref.)*
	Nein	0,919 [0,744–1,134]	0,941 [0,731–1,212]
*Bildung*	Kein Schulbesuch	1 *(Ref.)*	1 *(Ref.)*
	Grundschule	1,017 [0,716–1,444]	1,140 [0,723–1,798]
	Mittelschule	1,034 [0,755–1,418]	1,269 [0,839–1,920]
	Weiterführende/andere Schule	1,126 [0,827–1,532]	1,411 [0,942–2,113]
*PTS-Wert der Staatsangehörigkeit*	PTS-Wert 1–3	1 *(Ref.)*	1 *(Ref.)*
	PTS-Wert 4–5	**1,344 [1,019–1,771]**	**1,759 [1,217–2,542]**
*Postmigratorische Stressoren*			
*Aufenthaltsstatus*	Anerkennung	1 *(Ref.)*	1 *(Ref.)*
	Asylsuchend oder Ablehnung	**1,373 [1,128–1,671]**	**1,344 [1,062–1,701]**
*Anhörung*	Nein	1 *(Ref.)*	1 *(Ref.)*
	Ja	0,900 [0,735–1,103]	**0,710 [0,556–0,908]**
*Erwerbstätigkeit*	Erwerbstätig	1 *(Ref.)*	1 *(Ref.)*
	Nicht erwerbstätig	**1,610 [1,168–2,219]**	**1,483 [1,037–2,121]**
*Wohnzufriedenheit*	–	**0,907 [0,881–0,934]**	**0,943 [0,909–0,978]**
*Sprachkenntnisse (Deutsch)*	Gut	1 *(Ref.)*	1 *(Ref.)*
	Mittelmäßig	1,102 [0,844–1,438]	1,144 [0,841–1,557]
	Schlecht	1,299 [0,994–1,699]	1,239 [0,907–1,692]
*Einsamkeit*	–	**1,214 [1,179–1,249]**	**1,143 [1,103–1,184]**
*Psychosoziale Faktoren*			
*Selbstwertgefühl*	Hoch	–	1 *(Ref.)*
	Mittelmäßig bis gering	–	1,175 [0,900–1,533]
*Resilientes Copingverhalten*	Hoch	–	1 *(Ref.)*
	Mittelmäßig bis gering	–	**1,304 [1,038–1,639]**
*Lebenszufriedenheit*	Hoch	–	1 *(Ref.)*
	Mittelmäßig	–	0,910 [0,711–1,165]
	Gering	–	**1,996 [1,388–2,868]**
*Ängstlichkeit*	Keine Ängstlichkeit	–	1 *(Ref.)*
	Ängstlichkeit	–	**9,055 [7,236–11,331]**
*Nagelkerkes R*^2^	–	0,164	0,367
*N*	–	3288	2875

*OR* Odds Ratios verglichen mit der Referenzgruppe, *KI* 95 %-Konfidenzintervalle; *N* absolute Häufigkeiten der untersuchten Personen, Variationen in *N* resultieren aus fehlenden Werten

*Depressivität*: Patient Health Questionnaire‑2 (PHQ-2), keine Depressivität = Score 0–2, Depressivität = Score 3–6; *Partnerschaft*: Ja = verheiratet, Nein = ledig, geschieden, verwitwet; *Staatsangehörigkeit*: PTS-Wert 1–3 = Wert 1 bis 3 auf der Political Terror Scale, PTS-Wert 4–5: Wert 4 oder 5 auf der Political Terror Scale; *Aufenthaltsstatus*: Anerkennung = Flüchtlingsstatus, Asylberechtigte, anderer Schutzstatus, asylsuchend = Asylantrag wurde noch nicht entschieden, Ablehnung = Duldung, Ausreiseaufforderung; *Anhörung*: Ja = Anhörung des Asylantrags hat stattgefunden, Nein = Anhörung des Asylantrags hat noch nicht stattgefunden; *Erwerbstätigkeit*: erwerbstätig = Vollzeitbeschäftigung, Teilzeitbeschäftigung, Ausbildung oder Praktikum, nicht erwerbstätig = keine Erwerbstätigkeit; *Wohnzufriedenheit*: Skala von 0 (ganz und gar unzufrieden) bis 10 (ganz und gar zufrieden); *Einsamkeit*: Loneliness Scale-SOEP (LS-S); *Selbstwertgefühl*: „Ich habe eine positive Einstellung zu mir selbst“, hoch = Skalenwert 6–7, mittelmäßig = Skalenwert 3–5, gering = Skalenwert 1–2; *resilientes Copingverhalten*: Brief Resilient Coping Scale (BRCS), hoch = Score 17–20, mittelmäßig = Score 14–16, gering = Score 4–13; *Lebenszufriedenheit*: Lebenszufriedenheit‑1 (L-1), hoch = Skalenwert 7–10, mittelmäßig = Skalenwert 4–6, gering = Skalenwert 0–3; *Ängstlichkeit*: Generalized Anxiety Disorder‑2 (GAD-2), keine Ängstlichkeit = Score 0–2, Ängstlichkeit = Score 3–6; *fett gedruckte Zahlen*: Odds Ratios größer oder kleiner 1 mit einem Konfidenzniveau von 95 %; gewichtete Ergebnisse

Im Gesamtmodell weisen Alter, Geschlecht, Partnerschaft und Bildung keine signifikanten Zusammenhänge mit Depressivität auf. Dagegen haben Geflüchtete mit Staatsangehörigkeiten, die auf der Political Terror Scale (PTS) den beiden höchsten Werten 4 und 5 zugeordnet sind, eine 1,76-fach [1,22–2,54] höhere Chance für Depressivität als Geflüchtete aus Ländern mit niedrigeren PTS-Werten.

Mit Ausnahme der Prädiktorvariablen zu den Deutschkenntnissen zeigt das Gesamtmodell für alle einbezogenen postmigratorischen Stressoren statistisch signifikante Zusammenhänge mit Depressivität. Demnach haben Geflüchtete mit einem abgelehnten oder noch nicht entschiedenen Asylantrag eine 1,34-fach [1,06–1,70] höhere Chance für depressive Symptome als Geflüchtete mit einem anerkannten Asylantrag. Personen, bei denen eine offizielle Anhörung stattgefunden hat, haben eine geringere Chance für Depressivität (OR = 0,71 [0,56–0,91]). Dagegen ist Erwerbslosigkeit mit einer höheren Chance für depressive Symptome assoziiert (OR = 1,48 [1,04–2,12]). Es ist weiterhin erkennbar, dass sich die Chance für Depressivität mit zunehmender Wohnzufriedenheit verringert, jedoch in geringerem Ausmaß als in vorangehenden Modellen (OR = 0,94 [0,91–0,98]). Zusätzlich zeigt sich hinsichtlich der Einsamkeit, dass die Chance für depressive Symptome mit jeder Erhöhung um eine Einheit auf der LS‑S um das 1,14-Fache [1,10–1,18] ansteigt. Für die Prädiktoren Aufenthaltsstatus, Erwerbstätigkeit, Wohnzufriedenheit und Einsamkeit wurden in allen durchgeführten Berechnungen ausschließlich statistisch signifikante Werte ermittelt.

Das Selbstwertgefühl beschreibt den einzigen psychosozialen Faktor, der im Gesamtmodell keine statistische Signifikanz erreicht. Gegenüber Geflüchteten mit einem hohen resilienten Copingstil haben Geflüchtete, die ein mittelmäßiges bis geringes resilientes Copingverhalten aufweisen, eine höhere Chance für Depressivität (OR = 1,30 [1,04–1,64]). Personen mit einer geringen allgemeinen Lebenszufriedenheit haben im Vergleich zur Referenzgruppe eine doppelt so hohe Chance, depressiv zu sein (OR = 2,00 [1,39–2,87]). Pathologische Ängstlichkeit ist weiterhin statistisch signifikant mit depressiven Symptomen assoziiert. Auch im Gesamtmodell nimmt der Effektkoeffizient zur Ängstlichkeit den höchsten Wert an (OR = 9,06 [7,24–11,33]).

Nagelkerkes R^2^ zeigt, dass der Schätzerfolg durch das Gesamtmodell um 36,7 % verbessert werden kann (Tab. 5).

## Diskussion

Basierend auf den populationsbasierten, bundesweiten und repräsentativen Daten der IAB-BAMF-SOEP-Befragung von Geflüchteten 2016 konnte die Studie aufzeigen, dass verschiedene Faktoren der Postmigrationsphase deutliche Zusammenhänge mit dem Vorkommen depressiver Symptome bei erwachsenen Geflüchteten in Deutschland aufweisen. In dem adjustierten Gesamtmodell konnten Erwerbslosigkeit, Einsamkeit und ein abgelehnter oder noch nicht entschiedener Asylantrag als belastende Faktoren identifiziert werden, die die Chance für Depressivität erhöhen. Demgegenüber wirken eine stattgefundene Anhörung und eine höhere Wohnzufriedenheit protektiv und reduzieren die Chance für depressive Symptome.

In Übereinstimmung mit den Ergebnissen können auch verschiedene internationale Studien keine signifikanten Zusammenhänge zwischen dem Alter und depressiven Erkrankungen bei Geflüchteten nachweisen [26–28]. In anderen Studien stehen depressive Störungen mit einem jüngeren Alter [29–31] oder mit einem höheren Alter [8, 32] im Zusammenhang. Zusätzlich ist die Depression in Flüchtlingspopulationen häufig mit dem weiblichen Geschlecht assoziiert [8, 26, 28, 32, 33]. Dagegen konnten andere Studien ebenfalls keine signifikanten Geschlechterunterschiede ermitteln [13]. Möglicherweise sind Männer und Frauen ähnlichen Belastungen ausgesetzt, die sich gleichermaßen für beide Geschlechter auf die psychische Gesundheit auswirken [8]. In dieser Studie ist die Partnerschaft nicht mit depressiven Symptomen assoziiert, während sie in anderen Studien als ein protektiver Faktor für die Depression identifiziert wurde [26, 28]. In mehreren internationalen Untersuchungen steht die Bildung, wie auch im Gesamtmodell, nicht mit Depressivität im Zusammenhang [13, 28, 34]. Andere Studien weisen eine schlechtere psychische Gesundheit bei Geflüchteten mit einem höheren Bildungsstand nach. Soziale Degradierung und soziale Abstiegsprozesse im Zufluchtsland begünstigen das Auftreten psychischer Erkrankungen [32, 35, 36]. Vergleichbar mit den Ergebnissen der Studie zeigt auch der systematische Review von Lindert et al. (2018), dass Flüchtlinge aus Herkunftsländern mit massiven Menschenrechtsverletzungen und hohen Werten auf der Political Terror Scale (PTS) eine höhere psychosomatische Symptomatik aufweisen als Flüchtlinge aus Ländern ohne massive Menschenrechtsverletzungen [37].

Die Aufenthaltsgenehmigung ist in verschiedenen Studien mit einer reduzierten depressiven Symptomatik und einer verbesserten psychischen Gesundheit bei Geflüchteten assoziiert [27, 35, 38, 39]. Die niederländische Studie von Laban et al. (2004) zeigt, dass Asylsuchende mit einem langwierigen Asylverfahren im Vergleich zu Asylsuchenden mit einem kürzeren Asylverfahren eine höhere Chance für Depressionen, Angststörungen und somatoforme Störungen aufweisen [40]. Auch in der Studie von Poole et al. (2018) aus Griechenland ist die Dauer des Asylverfahrens mit depressiven Erkrankungen assoziiert [28]. Obwohl die Anhörung eine potenzielle Quelle der Aktualisierung traumatischer Erinnerungen ist und Traumafolgesymptome verschlimmern kann [41], stellt sie in den Analysen einen protektiven Faktor für Depressivität dar. Zu ähnlichen Ergebnissen kommt die Studie von Schock et al. (2014), in der Asylsuchende nach der Anhörung signifikant weniger depressive Symptome aufweisen [42]. Das Durchlaufen der Anhörung kann einen bedeutsamen Schritt in Richtung Sicherheit im Zufluchtsland bedeuten [41, 42]. Die Studie von Winkler et al. (2019) zeigt, dass eine subjektiv lang empfundene Wartezeit bis zur Anhörung und der Eindruck, in der Anhörung nicht alle asylrelevanten Details genannt zu haben, signifikant mit depressiver Symptomatik assoziiert sind [39]. Der positive Zusammenhang zwischen Erwerbslosigkeit und depressiven Symptomen bei Flüchtlingen wird durch zahlreiche internationale Studien bestätigt [8, 29, 35, 38]. Gesetzliche Restriktionen, geringe Sprachkenntnisse, Diskriminierung und die fehlende Anerkennung von Qualifikationen sind zentrale Barrieren für die Erwerbstätigkeit von Geflüchteten [35]. Hinsichtlich des protektiven Faktors der Wohnzufriedenheit zeigt eine Metaanalyse von Porter & Haslam (2005), dass Geflüchtete in dauerhaften privaten Unterkünften einen signifikant besseren psychischen Gesundheitszustand aufweisen als Geflüchtete in temporären institutionellen Unterkünften [32]. Durch fehlende Privatsphäre, enges Zusammenleben und eingeschränkte Handlungsmöglichkeiten beeinträchtigen Massenunterkünfte die psychische Gesundheit von Geflüchteten [9, 39]. Im Gegensatz zum Gesamtmodell können internationale Studien statistisch signifikante Zusammenhänge zwischen Depressivität und geringen Kenntnissen der Sprache des Aufnahmelandes nachweisen [8, 43]. Gemessen an der LS‑S weist die untersuchte Stichprobe mehr Einsamkeitsgefühle auf als die deutsche Allgemeinbevölkerung [44]. Einige internationale Studien belegen, dass eine geringe soziale Unterstützung mit erhöhter Depressivität bei Geflüchteten assoziiert ist [8, 31, 33, 38, 43, 45]. Teil eines sozialen Systems zu sein kann identitätsstiftend wirken und soziale Ressourcen bieten, die die Wahrscheinlichkeit für die Entwicklung von Hoffnungslosigkeit und Depression verringern [46].

Im Rahmen der Akkulturation können Geflüchtete unterschiedliche Belastungen erleben, die das Selbstwertgefühl reduzieren. Dazu gehören zum Beispiel Diskriminierung, Heimweh oder Gefühle der Entfremdung und Ausgrenzung. In der Studie von Park et al. (2018) haben nordkoreanische Flüchtlinge mit Suizidgedanken ein signifikant geringeres Selbstwertgefühl als diejenigen ohne Suizidgedanken [47]. Dagegen ist Depressivität im Gesamtmodell nicht statistisch signifikant mit dem Selbstwertgefühl assoziiert. Gemessen an der BRCS besitzt die untersuchte Stichprobe mit einem Mittelwert von 17,7 ein höheres resilientes Copingverhalten als die deutsche Allgemeinbevölkerungen, die Mittelwerte von 14,6 bei Frauen und 14,9 bei Männern aufweist [48]. Der signifikante negative Zusammenhang zwischen Resilienz und depressiven Symptomen wird durch internationale Studien bestätigt [45, 48]. Copingkompetenzen, Resilienzfaktoren und persönliche Ressourcen können die Ausprägung von Traumafolgestörungen minimieren und die Bewältigung unterstützen [41]. Die Mittelwerte der Kurzskala L‑1 zur Erfassung der allgemeinen Lebenszufriedenheit unterscheiden sich nicht deutlich zwischen der Stichprobe und der deutschen Allgemeinbevölkerung [21]. In Übereinstimmung mit den Ergebnissen von Park et al. (2017) zeigt das Gesamtmodell, dass eine geringe Lebenszufriedenheit mit erhöhter Depressivität assoziiert ist [31]. Auch der Mittelwert des GAD‑2 zur Messung pathologischer Ängstlichkeit ist mit 1,6 in der untersuchten Gesamtstichprobe gegenüber 0,8 in der deutschen Allgemeinbevölkerung deutlich erhöht [49]. Statistisch signifikante Zusammenhänge zwischen Ängstlichkeit und Depressivität sind auch in mehreren internationalen Studien gegeben. Insbesondere familienbezogene Ängste sind eng mit depressiven Symptomen assoziiert [33–35].

Der Mittelwert des PHQ‑2 zum Screening der Depressivität ist mit 1,5 in der untersuchten Gesamtstichprobe gegenüber 0,94 in einer repräsentativen Stichprobe der deutschen Allgemeinbevölkerung deutlich erhöht [49]. 19,4 % der befragten Geflüchteten weisen Depressivität auf. Dabei ist zu beachten, dass es sich um eine populationsbezogene Studie handelt und Geflüchtete mit schweren depressiven Störungen möglicherweise nicht erfasst wurden, da sie sich in psychotherapeutischer Behandlung befinden [37]. Populationsbasierte Stichproben weisen geringere Prävalenzen für psychische Störungen auf als institutionsbasierte Stichproben [12].

## Stärken und Limitationen

Eine zentrale Stärke der Studie ist die für das Bundesgebiet repräsentative Datengrundlage. Diese erlaubt belastbare Aussagen über Zusammenhänge hinsichtlich des psychischen Gesundheitszustands von Geflüchteten, die zwischen dem 01.01.2013 und dem 31.01.2016 nach Deutschland eingereist sind und einen formellen Asylantrag beim BAMF gestellt haben oder im Rahmen spezieller Programme aufgenommen wurden. Mit dem repräsentativen Sampling-Design wurden auch schutzsuchende Personen eingeschlossen, die den Asylantrag verzögert gestellt haben. Mehrsprachige persönliche Interviews, die etablierte Erhebungsinstrumente und innovative eingesprochene Audiodateien integrieren, wurden sowohl in Privathaushalten als auch in Erstaufnahmeeinrichtungen und Sammelunterkünften durchgeführt [14].

Neben diesen Stärken weist die Studie verschiedene Limitationen auf. Geflüchtete, die keinen Asylantrag gestellt haben, sind nicht in die Analysen eingeschlossen und bleiben unberücksichtigt. Der Ausschluss von Geflüchteten aufgrund von Sprachproblemen sowie die fehlende Verfügbarkeit der Erhebungsinstrumente in allen erforderlichen Sprachen können zu Verzerrungen führen. 41,1 % der Befragten, die die Sprachversion Deutsch/Englisch ausgewählt haben, kommen aus Russland, Georgien, Armenien, der Ukraine oder den Balkanstaaten (7 % der Stichprobe). Zu berücksichtigen ist, dass der Einsatz von Dolmetschern das Antwortverhalten der Befragten, insbesondere in Bezug auf persönliche Fragen zu postmigratorischen und psychosozialen Faktoren, beeinflusst haben kann [15, 17]. Zusätzlich konnten mit dem Studiendesign der Querschnittstudie Assoziationen berechnet werden, die keine Aussagen über kausale Beziehungen erlauben. Es ist davon auszugehen, dass innerhalb und zwischen den ausgewählten soziodemografischen, postmigratorischen und psychosozialen Faktoren Interdependenzen bestehen [35]. Es wurden allerdings keine Interaktionsterme berechnet, die potenzielle Wechselwirkungen zwischen den einzelnen Prädiktoren identifizieren. Zudem sind bei einzelnen Variablen spezifische Einschränkungen zu beachten. Die Variable zur Bildung bezieht sich auf den letzten Schulbesuch außerhalb von Deutschland. Sie enthält keine Informationen darüber, wie lange der Schulbesuch gedauert hat, ob die Schule mit einem Abschluss beendet wurde und in welchem Land die Schule besucht wurde. Es ist zu beachten, dass sich internationale Schulsysteme von dem deutschen System unterscheiden [50]. Die Zuordnung der Staatsangehörigkeiten zu den 5 Werten der Political Terror Scale (PTS) im Jahr 2016 ist mit mehreren Einschränkungen verbunden. Erstens können die PTS-Werte für die Staaten von Jahr zu Jahr variieren. Zweitens sind nicht alle Befragten im Jahr 2016 geflohen. Drittens muss das Land, aus dem die Befragten geflüchtet sind, nicht mit der Staatsangehörigkeit übereinstimmen. Außerdem könnte Ängstlichkeit einen intermediären Faktor darstellen, der einen Teil der Kausalkette zwischen Postmigrationsstressoren und Depressivität bildet. Die Aufnahme der Variablen zur Ängstlichkeit würde demnach eine Überadjustierung bewirken. Nach der Prüfung von Zusammenhangsmaßen zwischen Ängstlichkeit und Depressivität und in Übereinstimmung mit anderen internationalen Studien wurde die Ängstlichkeit jedoch als ein potenziell relevanter Prädiktor angesehen und in die Analysen integriert [33, 34].

## Fazit

Die untersuchten postmigratorischen Faktoren weisen nach der Adjustierung für soziodemografische und psychosoziale Faktoren statistisch signifikante und klinisch-epidemiologisch relevante Assoziationen mit Depressivität bei Geflüchteten auf. Während Erwerbslosigkeit, Einsamkeit und ein abgelehnter oder noch nicht entschiedener Asylantrag die Chance für depressive Symptome erhöhen, führen eine stattgefundene Anhörung und eine höhere Wohnzufriedenheit zu einer geringeren Chance für Depressivität.

Eine frühe Behandlung von psychischen Erkrankungen verhindert die Chronifizierung und ist von gesellschaftlicher Relevanz, da sie die Integrationsfähigkeit der Betroffenen fördert. Sektorenübergreifende sozialpolitische Maßnahmen, die die Stressoren der Postmigrationsphase berücksichtigen, können dazu beitragen, die psychische Krankheitslast in Flüchtlingspopulationen zu reduzieren und somit primärpräventive Wirkung entfalten. Dazu gehören neben der Optimierung rechtlicher Verfahren und dem Abbau gesetzlicher Einschränkungen auch Programme, die Flüchtlinge bei der Arbeitssuche und bei der sozialen Integration unterstützen. Die Anhörung sollte zeitnah und ohne lange Wartezeiten erfolgen, da sie Gefühle von Sicherheit und Erleichterung bewirken kann und somit das psychische Wohlbefinden fördert. Essenziell für die psychische Gesundheit ist zudem der uneingeschränkte Zugang zu einem gesundheitsfördernden Wohnumfeld, welcher in dauerhaften, privaten und stabilen Wohnunterkünften eher gegeben ist als in großen Sammelunterkünften.

Es sind weitere Studien erforderlich, die die Auswirkungen von belastenden und protektiven Faktoren der Postmigrationsphase auf die psychische Gesundheit von Geflüchteten prospektiv untersuchen, um kausale Pfade und Mechanismen abzubilden.

## Caption Electronic Supplementary Material


