# Supplementary material for: The effect of postmigration stressors on the prevalence of depressive symptoms among refugees in Germany. Analysis of the IAB-BAMF-SOEP Survey 2016
Source: Bundesgesundheitsblatt Gesundheitsforschung Gesundheitsschutz. 2020 Oct 25;63(12):1470–82. [Article in German] doi: 10.1007/s00103-020-03238-0 (PMC7686213; doi:10.1007/s00103-020-03238-0)
Supplement: Supplementary file 1 [file 103_2020_3238_MOESM1_ESM.pdf]

Bundesgesundheitsblatt 12/2020

Elektronisches Zusatzmaterial zum Beitrag:

Der Einfluss postmigratorischer Stressoren auf die Prävalenz depressiver Symptome bei Geflüchteten in Deutschland. Analyse anhand der IAB-BAMF-SOEP-Befragung 2016

Niklas Nutsch[1]
Kayvan Bozorgmehr[1,2*]

[1]Bevölkerungsmedizin und Versorgungsforschung, Fakultät für Gesundheitswissenschaften, Universität Bielefeld, Postfach 10 01 31, 33501 Bielefeld, Deutschland
[2]Sektion Health Equity Studies & Migration, Abteilung Allgemeinmedizin und Versorgungsforschung, Im Neuenheimer Feld 130.3, 69120 Heidelberg, Deutschland

Korrespondenzautor:
Prof. Dr. med. Kayvan Bozorgmehr (MSc)
AG2 - Bevölkerungsmedizin und Versorgungsforschung
Fakultät für Gesundheitswissenschaften
Universität Bielefeld
Postfach 10 01 31
33501 Bielefeld
Email: kayvan.bozorgmehr@uni-bielefeld.de

Inhalt:

Tabelle Z1: Modell I: Zusammenhang zwischen soziodemografischen Faktoren und Depression
Tabelle Z2: Modell II: Zusammenhang zwischen postmigratorischen Stressoren und Depression
Tabelle Z3: Modell III: Zusammenhang zwischen psychosozialen Faktoren und Depression
Tabelle Z4: Modell IV: Zusammenhang zwischen Depression und postmigratorischen Stressoren und soziodemografischen Faktoren
Tabelle Z5: Modell V: Zusammenhang zwischen Depression und postmigratorischen Stressoren, soziodemografischen Faktoren und psychosozialen Faktoren

Tabelle Z1: Modell I: Zusammenhang zwischen soziodemografischen Faktoren und Depression

**Prädiktorvariablen OR [95%KI]**		**Alter**	**Alter + Geschlecht**	**Alter + Geschlecht + Partnerschaft**	**Alter + Geschlecht + Partnerschaft + Bildung**	**Modell I final**
Alter		1,005 [0,998-1,012]	1,005 [0,998-1,012]	**1,012 [1,004-1,020]**	**1,009 [1,001-1,018]**	**1,010 [1,001-1,018]**
Geschlecht	Männlich		1 *(Ref.)*	1 *(Ref.)*	1 *(Ref.)*	1 *(Ref.)*
	Weiblich		0,984 [0,834-1,160]	1,078 [0.908-1,279]	1,078 [0,901-1,290]	1,053 [0,876-1,267]
Partnerschaft	Ja			1 *(Ref.)*	1 *(Ref.)*	1 *(Ref.)*
	Nein			**1,304 [1,099-1,548]**	1,170 [0,978-1,401]	1,188 [0,989-1,426]
Bildung	Kein Schulbesuch				1 *(Ref.)*	1 *(Ref.)*
	Grundschule				**0,672 [0,497-0,909]**	**0,661 [0,488-0,894]**
	Mittelschule				**0,674 [0,518-0,876]**	**0,680 [0,523-0,886]**
	Weiterführende/ Andere Schule				**0,743 [0,579-0,952]**	**0,713 [0,555-0,916]**
PTS-Wert der Staatsangehörigkeit	PTS-Wert 1-3					1 *(Ref.)*
	PTS-Wert 4-5					1,081 [0,857-1,363]
Nagelkerkes R^2		0,001	0,001	0,005	0,007	0,007
Modell-df		1	2	3	6	7
N		4134	4134	4111	3774	3711

Tabelle Z2: Modell II: Zusammenhang zwischen postmigratorischen Stressoren und Depression

**Prädiktorvariablen OR [95%KI]**		**Aufenthaltsstatus**	**Aufenthaltsstatus + Anhörung**	**Aufenthaltsstatus + Anhörung + Erwerbstätigkeit**	**Aufenthaltsstatus + Anhörung + Erwerbstätigkeit + Wohnzufriedenheit**	**Aufenthaltsstatus + Anhörung + Erwerbstätigkeit + Wohnzufriedenheit + Sprachkenntnisse**	**Modell II final**
Aufenthaltsstatus	Anerkennung	1 *(Ref.)*	1 *(Ref.)*	1 *(Ref.)*	1 *(Ref.)*	1 *(Ref.)*	1 *(Ref.)*
	Asylsuchend oder Ablehnung	**1,763 [1,516-2,050]**	**1,688 [1,434-1,987]**	**1,705 [1,448-2,008]**	**1,496 [1,264-1,770]**	**1,452 [1,225-1,720]**	**1,300 [1,087-1,556]**
Anhörung	Nein		1 *(Ref.)*	1 *(Ref.)*	1 *(Ref.)*	1 *(Ref.)*	1 *(Ref.)*
	Ja		0,902 [0,760-1,071]	0,892 [0,751-1,059]	0,862 [0,723-1,028]	0,867 [0,726-1,034]	0,933 [0,773-1,127]
Erwerbstätigkeit	Erwerbstätig			1 *(Ref.)*	1 *(Ref.)*	1 *(Ref.)*	1 *(Ref.)*
	Nicht erwerbstätig			**2,087 [1,582-2,751]**	**2,080 [1,569-2,759]**	**1,977 [1,486-2,630]**	**1,848 [1,365-2,501]**
Wohnzufriedenheit					**0,870 [0,849-0,892]**	**0,872 [0,851-0,894]**	**0,905 [0,881-0,930]**
Sprachkenntnisse (Deutsch)	Gut					1 *(Ref.)*	1 *(Ref.)*
	Mittelmäßig					**1,273 [1,010-1,605]**	1,161 [0,908-1,486]
	Schlecht					**1,397 [1,120-1,743]**	1,231 [0,971-1,559]
Einsamkeit							**1,207 [1,175-1,240]**
Nagelkerkes R^2		0,021	0,021	0,033	0,079	0,082	0,161
Modell-df		1	2	3	4	6	7
N		3906	3832	3832	3817	3813	3628

Tabelle Z3: Modell III: Zusammenhang zwischen psychosozialen Faktoren und Depression

**Prädiktorvariablen**<br>**OR [95%KI]**		**Selbstwertgefühl**	**Selbstwertgefühl + Resilientes Copingverhalten**	**Selbstwertgefühl + Resilientes Copingverhalten + Lebenszufriedenheit**	**Modell III final**
Selbstwertgefühl	Hoch	1 *(Ref.)*	1 *(Ref.)*	1 *(Ref.)*	1 *(Ref.)*
	Mittelmäßig bis gering	**1,639 [1,373-1,956]**	**1,482 [1,225-1,793]**	**1,312 [1,074-1,604]**	1,225 [0,971-1,545]
Resilientes Copingverhalten	Hoch		1 *(Ref.)*	1 *(Ref.)*	1 *(Ref.)*
	Mittelmäßig bis gering		**1,201 [1,018-1,417]**	1,131 [0,951-1,344]	**1,226 [1,005-1,495]**
Lebenszufriedenheit	Hoch			1 *(Ref.)*	1 *(Ref.)*
	Mittelmäßig			**1,752 [1,467-2,093]**	**1,418 [1,157-1,737]**
	Gering			**6,420 [5,035-8,187]**	**3,322 [2,498-4,417]**
Ängstlichkeit	Keine Ängstlichkeit				1 *(Ref.)*
	Ängstlichkeit				**9,799 [8,123-11,821]**
Nagelkerkes R^2		0,011	0,011	0,105	0,330
Modell-df		1	2	4	5
N		3881	3549	3544	3493

Tabelle Z4: Modell IV: Zusammenhang zwischen Depression und postmigratorischen Stressoren und soziodemografischen Faktoren

**Prädiktorvariablen**<br>**OR [95%KI]**		**Modell I +**<br>**Aufenthaltsstatus**	**Modell I +**<br>**Aufenthaltsstatus + Anhörung**	**Modell I +**<br>**Aufenthaltsstatus + Anhörung + Erwerbstätigkeit**	**Modell I +**<br>**Aufenthaltsstatus + Anhörung + Erwerbstätigkeit + Wohnzufriedenheit**	**Modell I +**<br>**Aufenthaltsstatus + Anhörung + Erwerbstätigkeit + Wohnzufriedenheit + Sprachkenntnisse**	**Modell IV final**
**Soziodemografische Faktoren**							
Alter		**1,009 [1,001-1,018]**	**1,009 [1,001-1,018]**	**1,009 [1,001-1,018]**	**1,013 [1,004-1,022]**	**1,011 [1,002-1,020]**	1,006 [0,997-1,016]
Geschlecht	Männlich	1 *(Ref.)*	1 *(Ref.)*	1 *(Ref.)*	1 *(Ref.)*	1 *(Ref.)*	1 *(Ref.)*
	Weiblich	0,982 [0,809-1,192]	0,952 [0,782-1,158]	0,900 [0,738-1,096]	0,960 [0,784-1,175]	0,942 [0,769-1,154]	1,088 [0,877-1,350]
Partnerschaft	Ja	1 *(Ref.)*	1 *(Ref.)*	1 *(Ref.)*	1 *(Ref.)*	1 *(Ref.)*	1 *(Ref.)*
	Nein	1,003 [0,829-1,213]		1,004 [0,828-1,217]	0,948 [0,779-1,154]	0,963 [0,791-1,172]	0,919 [0,744-1,134]
Bildung	Kein Schulbesuch	1 *(Ref.)*	1 *(Ref.)*	1 *(Ref.)*	1 *(Ref.)*	1 *(Ref.)*	1 *(Ref.)*
	Grundschule	0,793 [0,581-1,084]	0,825 [0,601-1,133]	0,873 [0,635-1,200]	0,836 [0,605-1,157]	0,847 [0,612-1,172]	1,017 [0,716-1,444]
	Mittelschule	0,761 [0,579-1,001]	0,765 [0,579-1,011]	0,792 [0,599-1,047]	0,756 [0,567-1,007]	0,789 [0,591-1,053]	1,034 [0,755-1,418]
	Weiterführende/ Andere Schule	0,844 [0,649-1,098]	0,848 [0,649-1,108]	0,902 [0,689-1,180]	0,850 [0,646-1,119]	0,929 [0,701-1,232]	1,126 [0,827-1,532]
PTS-Wert der Staatsangehörigkeit	PTS-Level 1-3	1 *(Ref.)*	1 *(Ref.)*	1 *(Ref.)*	1 *(Ref.)*	1 *(Ref.)*	1 *(Ref.)*
	PTS-Level 4-5	1,226 [0,961-1,564]	1,209 [0,945-1,547]	1,170 [0,913-1,499]	**1,318 [1,018-1,706]**	1,286 [0,993-1,666]	**1,344 [1,019-1,771]**
**Postmigratorische Stressoren**							
Aufenthaltsstatus	Anerkennung	1 *(Ref.)*	1 *(Ref.)*	1 *(Ref.)*	1 *(Ref.)*	1 *(Ref.)*	1 *(Ref.)*
	Asylsuchend oder Ablehnung	**1,786 [1,507-2,116]**	**1,696 [1,416-2,030]**	**1,702 [1,421-2,040]**	**1,519 [1,261-1,1828]**	**1,479 [1,227-1,782]**	**1,373 [1,128-1,671]**
Anhörung	Ausstehend		1 *(Ref.)*	1 *(Ref.)*	1 *(Ref.)*	1 *(Ref.)*	1 *(Ref.)*
	Stattgefunden		0,867 [0,721-1,043]	0,857 [0,712-1,031]	**0,813 [0,673-0,983]**	**0,824 [0,681-0,997]**	0,900 [0,735-1,103]
Erwerbstätigkeit	Erwerbstätig			1 *(Ref.)*	1 *(Ref.)*	1 *(Ref.)*	1 *(Ref.)*
	Nicht erwerbstätig			**2,032 [1,510-2,732]**	**1,980 [1,463-2,680]**	**1,882 [1,387-2,556]**	**1,610 [1,168-2,219]**
Wohnzufriedenheit					**0,847 [0,847-0,894]**	**0,874 [0,851-0,898]**	**0,907 [0,881-0,934]**
Sprachkenntnisse (Deutsch)	Gut					1 *(Ref.)*	1 *(Ref.)*
	Mittelmäßig					1,220 [0,950-1,567]	1,102 [0,844-1,438]
	Schlecht					**1,413 [1,100-1,813]**	1.299 [0,994-1,699]
Einsamkeit							**1,214 [1,179-1,249]**
Nagelkerkes R²		0,025	0,025	0,036	0,081	0,085	0,164
Modell-df		8	9	10	11	13	14
N		3515	3457	3457	3444	3442	3288

Tabelle Z5: Modell V: Zusammenhang zwischen Depression und postmigratorischen Stressoren, soziodemografischen Faktoren und psychosozialen Faktoren

**Prädiktorvariablen**<br>**OR [95%KI]**		**Modell IV + Selbstwertgefühl**	**Modell IV + Selbstwertgefühl + Resilientes Copingverhalten**	**Modell IV + Selbstwertgefühl + Resilientes Copingverhalten + Lebenszufriedenheit**	**Modell V**
**Soziodemografische Faktoren**					
Alter		1,007 [0,997-1,017]	1,009 [0,999-1,019]	1,009 [0,999-1,020]	1,004 [0,992-1,015]
Geschlecht	Männlich	1 *(Ref.)*	1 *(Ref.)*	1 *(Ref.)*	1 *(Ref.)*
	Weiblich	1,082 [0,866-1,352]	1,078 [0,855-1,358]	1,164 [0,921-1,470]	0,853 [0,653-1,115]
Partnerschaft	Ja	1 *(Ref.)*	1 *(Ref.)*	1 *(Ref.)*	1 *(Ref.)*
	Nein	0,908 [0,731-1,126]	0,900 [0,720-1,125]	0,878 [0,701-1,101]	0,941 [0,731-1,212]
Bildung	Kein Schulbesuch	1 *(Ref.)*	1 *(Ref.)*	1 *(Ref.)*	1 *(Ref.)*
	Grundschule	0,904 [0,631-1,295]	0,935 [0,632-1,383]	0,991 [0,666-1,474]	1,140 [0,723-1,798]
	Mittelschule	0,934 [0,677-1,290]	1,085 [0,764-1,542]	1,100 [0,769-1,574]	1,269 [0,839-1,920]
	Weiterführende/ Andere Schule	1,096 [0,802-1,498]	1,301 [0,925-1,830]	1,298 [0,916-1,839]	1,411 [0,942-2,113]
PTS-Wert der Staatsangehörigkeit	PTS-Wert 1-3	1 *(Ref.)*	1 *(Ref.)*	1 *(Ref.)*	1 *(Ref.)*
	PTS-Wert 4-5	**1,593 [1,189-2,136]**	**1,583 [1,146-2,187]**	**1,624 [1,169-2,254]**	**1,759 [1,217-2,542]**
**Postmigratorische Stressoren**					
Aufenthaltsstatus	Anerkennung	1 *(Ref.)*	1 *(Ref.)*	1 *(Ref.)*	1 *(Ref.)*
	Asylsuchend oder Ablehnung	**1,419 [1,160-1,737]**	**1,450 [1,177-1,786]**	**1,393 [1,127-1,722]**	**1,344 [1,062-1,701]**
Anhörung	Nein	1 *(Ref.)*	1 *(Ref.)*	1 *(Ref.)*	1 *(Ref.)*
	Ja	0,914 [0,742-1,126]	0,920 [0,740-1,143]	0,893 [0,716-1,113]	**0,710 [0,556-0,908]**
Erwerbstätigkeit	Erwerbstätig	1 *(Ref.)*	1 *(Ref.)*	1 *(Ref.)*	1 *(Ref.)*
	Nicht erwerbstätig	**1,573 [1,137-2,176]**	**1,627 [1,160-2,264]**	**1,600 [1,145-2,236]**	**1,483 [1,037-2,121]**
Wohnzufriedenheit		**0,908 [0,882-0,936]**	**0,910 [0,888-0,945]**	**0,939 [0,908-0,970]**	**0,943 [0,909-0,978]**
Sprachkenntnisse (Deutsch)	Gut	1 *(Ref.)*	1 *(Ref.)*	1 *(Ref.)*	1 *(Ref.)*
	Mittelmäßig	1,118 [0,852-1,465]	1,110 [0,842-1,462]	1,111 [0,841-1,468]	1,144 [0,841-1,557]
	Schlecht	1,225 [0,932-1,611]	1,195 [0,904-1,578]	1,159 [0,875-1,534]	1,239 [0,907-1,692]
Einsamkeit		**1,206 [1,171-1,243]**	**1,217 [1,180-1,256]**	**1,194 [1,157-1,232]**	**1,143 [1,103-1,184]**
**Psychosoziale Faktoren**					
Selbstwertgefühl	Hoch	1 *(Ref.)*	1 *(Ref.)*	1 *(Ref.)*	1 *(Ref.)*
	Mittelmäßig bis gering	**1,422 [1,146-1,764]**	**1,291 [1,026-1,626]**	1,261 [0,996-1,595]	1,175 [0,900-1,533]
Resilientes Copingverhalten	Hoch		1 *(Ref.)*	1 *(Ref.)*	1 *(Ref.)*
	Mittelmäßig bis gering		1,135 [0,929-1,386]	1,145 [0,935-1,404]	**1,304 [1,038-1,639]**
Allgemeine Lebenszufriedenheit	Hoch			1 *(Ref.)*	1 *(Ref.)*
	Mittelmäßig			1,155 [0,929-1,436]	0,910 [0,711-1,165]
	Gering			**3,293 [2,398-4,522]**	**1,996 [1,388-2,868]**
Ängstlichkeit	Keine Ängstlichkeit				1 *(Ref.)*
	Ängstlichkeit				**9,055 [7,236-11,331]**
Nagelkerkes R^2		0,161	0,164	0,189	0,367
Modell-df		15	16	18	19
N		3137	2915	2912	2875
